# Experiences of South African caregivers disclosing to their children living with HIV: Qualitative investigations

**DOI:** 10.1371/journal.pone.0277202

**Published:** 2022-11-29

**Authors:** Celeste Joyce, Candice Ramsammy, Lisa Galvin, Given Leshabane, Afaaf Liberty, Kennedy Otwombe, Janice Buckley, Minja Milovanovic, Avy Violari

**Affiliations:** 1 Perinatal HIV Research Unit, Faculty of Health Sciences, University of the Witwatersrand, Johannesburg, South Africa; 2 Department of Psychiatry, Faculty of Health Sciences, University of the Witwatersrand, Johannesburg, South Africa; 3 School of Public Health, Faculty of Health Sciences, University of the Witwatersrand, Johannesburg, South Africa; 4 African Potential Management Consultancy, Kyalami, South Africa; Emory University School of Medicine, UNITED STATES

## Abstract

Awareness of Human Immunodeficiency Virus (HIV) status improves health outcomes in children living with HIV, yet caregivers often delay disclosure. This qualitative investigation explored, through observation, how 30 caregivers responded to a HIV Disclosure study conducted between 2017 and 2020 at Chris Hani Baragwanath Academic Hospital, Soweto, South Africa. Caregivers were assisted in disclosing to their children, aged 7–13 years; followed by a sub-sample of caregivers providing in-depth interviews to elaborate on findings.1) Barriers to disclosure included: caregivers being ill equipped, the fear of negative consequences and children considered lacking emotional or cognitive readiness. 2) Deflecting diagnosis from their children and the need for medication, motivated caregivers to disclosure. 3) Apprehension was evident during disclosure; however, overall disclosure was a positive experience with the support of the healthcare providers. These results highlight the significant role healthcare providers’ play in supporting caregivers through the disclosure process.

## Introduction

The increase in Antiretroviral Therapy (ART) coverage led to a decline in HIV related deaths of children in Southern Africa, now making Human Immunodeficiency Virus (HIV) a chronic condition that requires comprehensive multidisciplinary care [1–10]. Disclosing HIV status to children who were perinatally-infected remains a challenge despite studies suggesting that it improves health and emotional outcomes [2, 3, 8–15]. Disclosure is often done at the discretion of the healthcare providers and is reliant on the resources and guidelines available [12]. The South African Department of Health (NDoH) [16] suggests that partial disclosure takes place from 3 to 9 years of age, and full disclosure is to take place from 10 years of age. The World Health Organisation (WHO) [4] suggests disclosing to school going children between 6 and 12 years of age [16]. Both guidelines recommend a comprehensive approach to disclosure and that pre-disclosure (i.e. explaining the disclosure process to the caregiver) is not completed in front of the child so that the child’s well-being is safeguarded [4, 16]. Despite these detailed guidelines, many children are unaware of their HIV status and their caregivers are hesitant to disclose to them [17].

It is perhaps reasonable to expect the parents’ hesitancy as disclosure has far reaching psychosocial implications [18]. Caregivers and their children infected with HIV live as a part of a greater “whole”. In accordance with Social Learning Theory, an individual is a member of his/her immediate family, extended family, culture, peer group, school and country [19, 20]. Not only do they influence their environment but they are directly influenced by the attitudes, perspectives and behaviours of others [19, 20]. Caregiver attitudes towards HIV and disclosure are shaped by their personal experiences within the environment and the attitudes of those around them. This shapes when and how disclosure is addressed in their own families [15, 20, 21]. Several barriers to disclosure have been identified, including: social stigma; fear of discrimination; the impact on the child’s emotional/psychological well-being; fear of the child’s resentment; untimely disclosure of the child’s and/or the caregiver’s HIV status to others; exposure of the caregiver’s “personal secrets”. There is less known about the reported negative implications including sadness, depression, stigmatisation and behavioural problems [22, 23].

Lack of knowledge on how and when to approach the disclosure question is another important factor delaying disclosure [4, 13, 16–19, 24–27]. A study in Ghana [26] and another in Peru [28], reported that few mothers were confident in their ability to disclose their status due to social stigma and the possibility of this stigma being passed onto their children. The VUKA (meaning “Let’s wake up” in isiZulu) study, which focused on pre-adolescent children who were aware of their HIV status and that of their family members, emphasised the importance of family involvement in mental health and health promotion interventions [29]. Therefore, interventions aimed at assisting mothers disclose their child’s HIV status to their children in a developmentally appropriate manner, i.e. at a level appropriate to their understanding and emotional maturity, could improve the mother’s confidence in disclosing her own status. Therefore, we developed a study that guided caregivers through a gradual disclosure process and explored their responses and interactions with their children before and after disclosure. Psychometric investigations assessed the impact on the child’s emotional state and adaptive behaviours and are reported elsewhere [30].

Here we present the caregivers’ motivation for and response to each stage of the disclosure process. As the caregiver is at the core of the disclosure or non-disclosure issue, it is important to understand their responses and perceptions and use these to shape future interventions.

## Methods

### Study setting and participants

The study was conducted between 2017 and 2020 at the Perinatal HIV Research Unit (PHRU), Chris Hani Baragwanath Academic Hospital, Soweto, South Africa. Children between the ages 7–13 years, attending the Wellness clinic at PHRU with documented perinatal HIV status and who had not been previously fully disclosed to, were eligible and were recruited along with their caregivers (30 dyads).

Caregivers were eligible if they were the child’s biological parent, legal guardian, foster parent, or another person responsible for the protection and promotion of the child’s health and well-being. Following the completion of the main study in 2020 (between 9 and 15 months after full-disclosure), caregivers were contacted telephonically and invited to participate in the semi-structured in-depth interviews where they shared their perspective of the disclosure program. These participants were purposely sampled to ensure the capture of a broad range of experiences from both female and male perspectives, as well as the perspectives of one caregiver who discontinued the study. Invitations were overextended to the 30 participants with a view of reaching saturation after at least 20 interviews and anticipating refusals. However, none of the invitations to participate were refused.

### Study procedures

**Fig 1:**
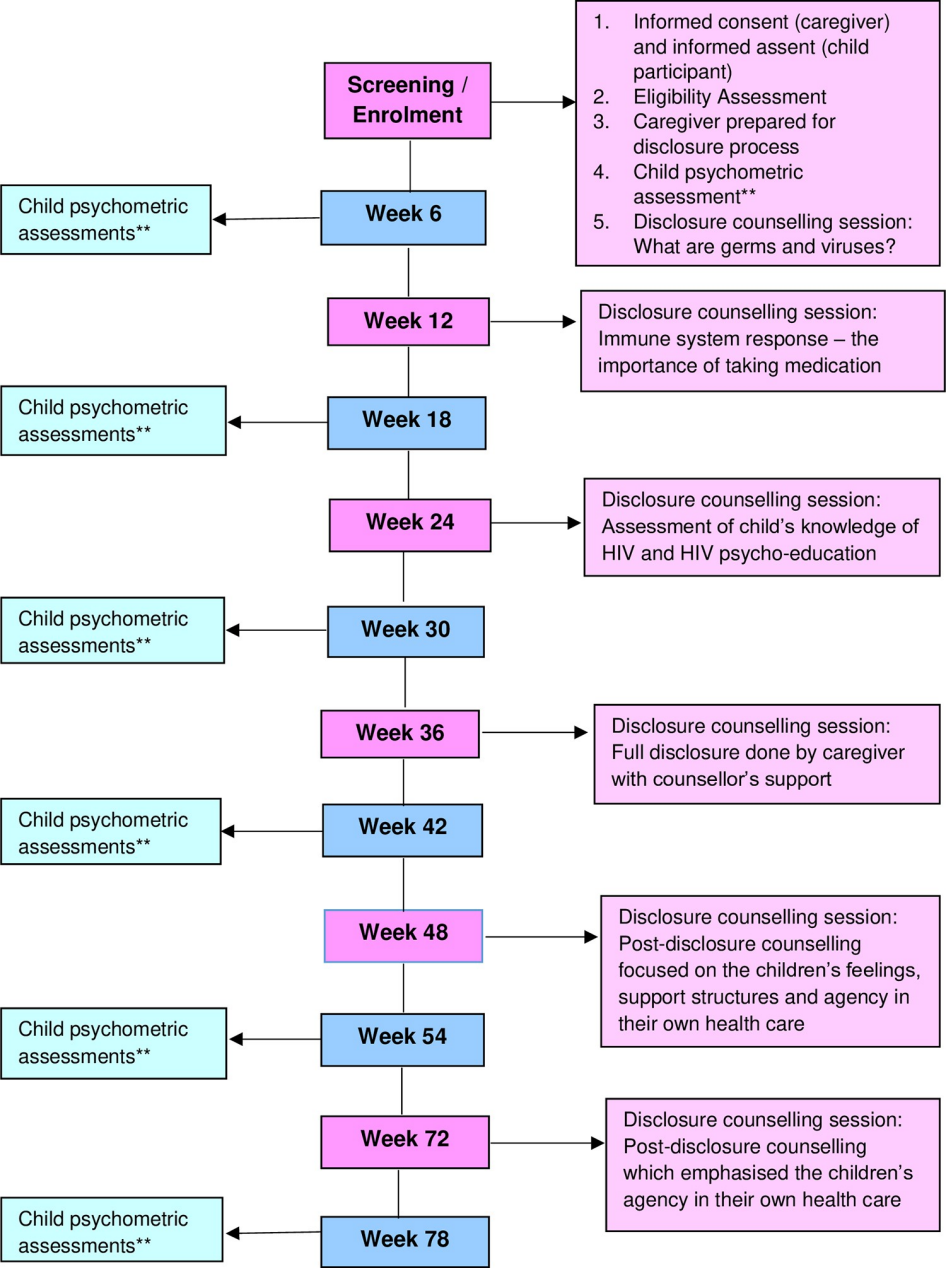
The disclosure study visit guideline over 78 weeks*. *This is a guideline for counselling visits that were flexible and allowed for acceleration and deceleration. **Psychological assessments completed by child participants.

Study visits took place over 78 weeks, alternating six weekly between disclosure counselling sessions (completed week 72) and psychometric sessions (completed week 78) (Fig 1). The counselling sessions were led by two female healthcare providers (i.e. a HIV counsellor/nurse and social worker), who were part of the study team. They were trained by the study Principal Investigator to use the “Right to Care Mini Flipster” [31] and the United States Agency for International Development (USAID) / U.S. President’s Emergency Plan for AIDS Relief (PEPFAR) [32–34] disclosure material, as well as in reporting their observations on the Disclosure Counselling Forms (DCF).

The initial pre-disclosure session took place in a private room and was attended by the caregiver without the child present. During this session, the caregiver was provided with information and prepared for the disclosure process. The child and the caregiver participated together in the six disclosure sessions that followed. Fidelity was ensured through healthcare worker training on the disclosure tools and counselling processes and the consistency of the counselling and assessment program with regard to tools and administration. Despite following the process consistently, some children were not able to receive full disclosure. The healthcare provider facilitated each session, which lasted for between 45 minutes and an hour.

The initial sessions provided education about health, the immune system and the importance of medication adherence. The following sessions then assessed the child’s knowledge about HIV and with the revision of unclear concepts. Based on the child’s comprehension of these sessions, full disclosure was done by the caregiver with the support of the healthcare provider. Post-disclosure counselling sessions focused on children’s feelings, made them aware of their support structures, and emphasised their own agency in their care. The process was individualised and flexible, allowing for additional visits, revisiting of topics, and accelerating or decelerating the process.

In-depth interviews were conducted with 15 of the caregivers in a private office at the research facility.

### Tools

#### Counselling tools

This study provided standardised and developmentally appropriate disclosure counselling using the “Right to Care” Disclosure Tool and the United States Agency for International Development (USAID) / U.S. President’s Emergency Plan for AIDS Relief (PEPFAR) disclosure booklets [31–34]. The “Right to Care” Disclosure tool [32] consists of two developmentally appropriate Flipster books: “Preparing Children for Pre-Disclosure Through Play” utilises stories, activities and songs to teach the younger child, below 12 years of age, about germs, healthy living and illness; and “Mini-Flipster: Disclosure Tool for Adolescents Ages 12 & Up” explores the topics of Pre-Disclosure, Full-Disclosure and Post-Disclosure using the Socratic method of questioning. The USAID/PEPFAR Disclosure Booklets [32–34] also address pre-disclosure, full-disclosure and post-disclosure using stories and the Socratic method of questioning. Both tools were developed to assist caregivers to disclose with the guidance of healthcare providers and were used together in this study.

#### Disclosure Counselling Form (DCF)

The DCFs were specifically developed by the research team to record disclosure progression, provide progress notes and information regarding who accompanied the child to each session. Healthcare providers were trained in how to complete the DCF with their observations. After each counselling session, throughout each stage of the disclosure, the healthcare provider recorded their detailed observations regarding the caregiver’s, child’s and the dyads’ interaction, and other relevant information provided by the caregiver during the session. The DCF was used to maintain structure and to identify any psycho-social stressors which could be addressed immediately through further counselling, medical or social work intervention.

### Data collection

Data was collected in two phases; during the disclosure process and during the in depth interviews that occurred after end of the main study.

#### Healthcare provider’s observations of the counselling sessions

The healthcare providers noted their observations of child-caregiver interactions and reactions on the DCF after each disclosure counselling session for each participant (6 sessions).

#### In-depth interviews

There is little information in the literature regarding caregivers’ reactions to the disclosure process. DCF information was identified as being subjective and from the perspective of the healthcare provider. Therefore, it was felt that it would be pertinent to include the semi-structured in-depth interviews as they explored specific themes more deeply. The research team developed an in-depth, semi-structured, interview guide. Open-ended questions were used to elicit the perspectives of the caregivers on the disclosure process. The interviews were developed and formulated with the purpose of understanding the challenges and concerns caregivers had around disclosing to their children before joining the disclosure study; the disclosure process itself; and to identify any concerns and parenting challenges that may arise in the future. Two of the authors conducted these interviews, a registered counsellor, who had previously had minimal contact with the caregivers, and research psychologist, who was not involved in the main study. The interviews took approximately 30–40 minutes to complete. Interviewers were proficient in counselling and conducting interviews. They received training on the interview schedule and were fluent in the spoken local languages of the participants and English. As they were also familiar with the participants’ culture, it was unlikely that cultural barriers would have skewed participant answers.

Caregivers were interviewed primarily in English. However, questions were clarified and participants were able to respond in their preferred language (i.e. English, isiZulu or seSotho) and with their consent, audio recordings were made. The recordings were later transcribed and reviewed by the research team. All transcriptions were translated into English and reviewed before analysis.

### Data analysis

**Fig 2:**
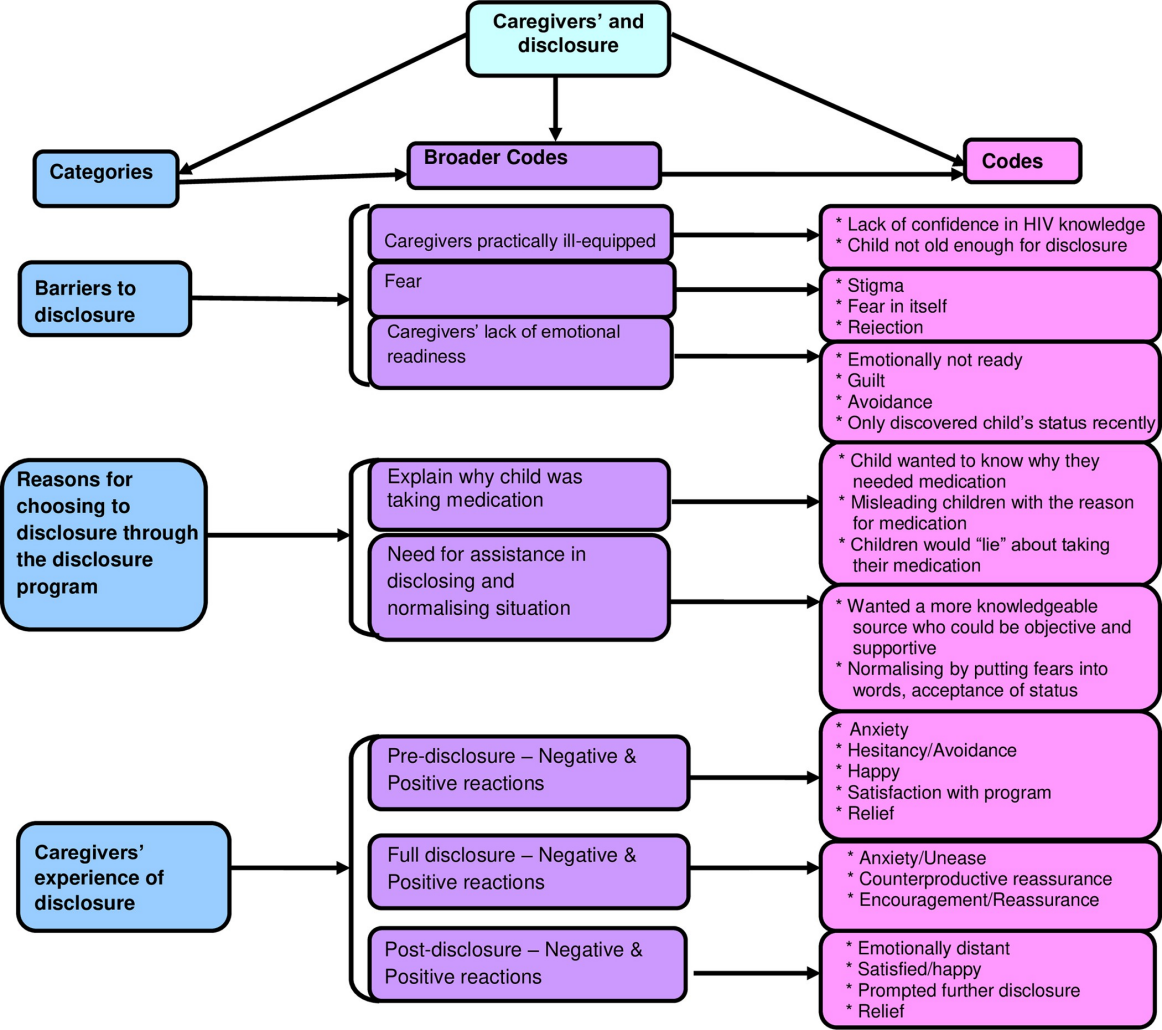
Coding tree. Four female researchers (a Clinical Psychologist, Psychiatrist, Research Psychologist and Registered Counsellor) independently reviewed and familiarised themselves with an average of 6 DCFs for each participant, as well as the 15 interview transcripts. The researchers represented different cultural backgrounds and ages, and counselling fields and areas of expertise. They were experienced in interpreting subtle emotional nuances in responses, ensuring trustworthiness, credibility and reliability in the data interpretation. This assisted with improving the likelihood that cultural or training bias would unduly influence perceptions of results when coding data. Furthermore, none of the researchers were counsellors in the study, reducing the likelihood of bias when interpreting the data.

Information regarding barriers to disclosure, as well as observations relating to the caregiver and their response to the intervention from both the DCF’s and in-depth interview transcriptions were analysed manually using content analysis. The analysis included the development of a code book by establishing specific categories, broader codes and codes (Fig 2), reviewing of the codes, coding and interpreting the data in order to describe, compare, categorise and conceptualise [35, 36].

Initially, researchers worked individually to identify categories within the DCFs. These identified categories were discussed amongst the researchers and once agreed upon, the codebook was developed. The tabulation of categories was developed in Microsoft Word. The broader codes were identified and data was then grouped together into codes. These were tabulated in the same Microsoft Word document accompanied by extracts from the DCFs according to their broader codes and codes. These broader codes and codes were again compared across researchers, merging commonalities. Discrepancies were discussed until consensus was reached. The categories, broader codes and codes were used to re-evaluate the data and to quantify the frequency of codes noted. The in-depth interview transcripts analysis used the same method as the DCF’s, after analysis of the DCFs were completed. Similarities and differences between the two sets of data were identified and combined to provide a more complete understanding of the caregivers’ reasons for not disclosing to their children before joining the program.

Caregiver demographics were analysed using descriptive statistics.

### Ethics/Protection of human subjects

Ethical approval was obtained from the University of the Witwatersrand Human Research Ethics Committee (HREC, reference number 170107) in Johannesburg. Informed consent was obtained from all caregivers. Assent was obtained from all children in their preferred language. The assent process followed accepted international guidelines which respect the child’s decision to refuse participation. Informed consent and consent to audio-recording was obtained from all caregivers who participated in the in-depth interviews. Participant confidentiality was maintained through the use of participant identification numbers.

## Results

Thirty child and caregiver dyads were enrolled into the study (See Table 1). The median age of the caregivers was 39 years (IQR = 34–49). The majority of the caregivers (90%) were biological parents and predominantly mothers (24 mothers vs 3 fathers), and the remaining 3 were grandmothers or aunts. However, during the study, one father was unable to continue and the mother accompanied the child; a second participant’s mother was unable to complete the study and the father attended the final counselling session. In both cases, this was due to employment commitments. Twenty-eight caregivers were HIV-infected themselves and 27 were receiving ART. The majority of the adults were unemployed (67%) and had completed a secondary level of education (83%).

**Table 1:** Caregiver demographics.

**Relationship to participant**	Mother (%)	23 (77)
	Father (%)	4 (13)
	Other (%)	3 (10)
**HIV Status**	Positive (%)	28 (93)
	Negative (%)	2 (07)
**Receiving ART**	Yes (%)	27 (96)
	No (%)	1 (04)
**Education Level**	Post Grade 12 (%)	3 (10)
	Secondary (%)	1 (03)
	Primary (%)	25 (85)
	Unknown (%)	1 (03)
**Employment Status**	Employed (%)	10 (33)
	Unemployed (%)	20 (67)

Two children did not complete the study, both never receiving full disclosure. One child’s caregiver was no longer contactable after week 24. The other child’s caregiver was unavailable after week 48 but did consent to participating in the in-depth interviews.

Of the 15 caregivers who participated in the in-depth interviews, the majority were mothers (13 mothers vs 2 fathers) with a median age of 38 years (IQR = 34–43). Thirteen of 15 caregivers had fully disclosed to their children by the end of the study. One caregiver completed all study visits but due to child’s young age and insufficient concept comprehension, the child did not receive full disclosure. The remaining dyad discontinued the study prior to full disclosure, as the caregiver was no longer available to attend study visits.

Three central themes, related to the caregivers’ experience of disclosure, emerged during analysis: their barriers towards disclosure; their reason for disclosing through the study; and the caregivers’ response to disclosure.

### 1. The caregivers’ reservations to disclosure of the child’s HIV status

Caregivers were asked at the first disclosure counselling session what had prevented them from disclosing their child’s HIV status to them. This open-ended question was identified as a central theme during the content analysis. These reasons were further supported by the in-depth interviews. Three sub-themes were identified:

1.1 Caregiver practically ill equipped to disclose
1.2 Fear
1.3 Lack of emotional readiness

#### 1.1 Caregiver practically ill equipped for disclosure

The most common reason for not disclosing (43%) feeling that the children were still too young for disclosure and that younger children would not be able to understand HIV/AIDS. There was, however, no reference to a particular age at which the child would be “ready” or why they thought the child would not understand the information. The comments around younger children not being ready to be disclosed to seemed to reflect the caregivers’ own insecurities and inadequacies in their ability to disclose to their children, which was reported by the caregiver during the initial disclosure sessions, more so than concerns around appropriate age for disclosure.

The In-depth interviews explored appropriate age for disclosure further and caregivers gave age ranges between 11–18 years as an appropriate age for disclosure, as they would have a better understanding of the seriousness of HIV and the implications for their lives.

Almost three quarters (70%) of caregivers felt ill equipped to disclose, citing challenges in communicating with their children about their illness. Eleven caregivers (37%) reported that a lack of confidence in their own HIV knowledge restricted their ability to disclose to their children. Some caregivers did not know what and how much information to share, and how much detail to include about the illness. Others were concerned that they were uncertain of their ability to respond to and/or handle questions that would arise after disclosure. During the in-depth interviews, four of the 15 caregivers elaborated on this lack of confidence in their HIV knowledge.

*“My problem was that I was not going to be able to face the questions that would arise and did not have a plan as to how I go about starting the conversation with him” (caregiver of participant 018*, *interview*).

#### 1.2 Fear

Fear was a common reason for not disclosing in 40% of the caregivers, which the in-depth interviews confirmed. There were equal reports of fear of stigma, rejection from their child and fear of negative consequences.

Ten caregivers expressed fear of stigma. Five caregivers (50%) had not disclosed prior to the study as they were concerned about how others would react to the child should they disclose their status Two caregivers (20%) expressed concerns about how family would react to finding out that the child was HIV positive. The other three caregivers (30%) were afraid of the child being unable to maintain confidentiality (“keep the secret”) about their status.

Fear of social stigma was also evident during the in-depth interviews where one of the caregivers stated:

“*We were scared and afraid that he would go about talking about it in the neighbourhood*. *Scared of being stigmatised” (caregiver of participant 028*, *interview*).

Furthermore, five caregivers (17%) cited a fear of rejection from their children after disclosure. During the in-depth interviews, this fear of rejection was not only limited to the caregiver being rejected by the child but also to the child being rejected by other family members

“*I was scared to tell him he is HIV+ because it is only him that is infected … I thought that if the other two knew he was HIV they might distance themselves from him and not allow him to be part of them” (caregiver of participant 031*, *interview*).

Three caregivers (10%) displayed generalised fear, related to how the child would react to disclosure (i.e. running away or responding negatively to the news). Two other caregivers (7%) were afraid to broach the subject of HIV and disclosure. This was reiterated during the in-depth interviews where the caregivers spoke of being afraid to broach the subject and one expressed concern that disclosing to the child would lead to worse consequences.

“*How would I explain to him what is HIV*? *And what if he gets angry and kills himself” (caregiver of participant 005*, *Interview*).

#### 1.3 Lack of emotional readiness

Lack of the caregivers’ emotional readiness to disclose to their children was expressed in their feelings of guilt, anxiety, avoidance and emotional unpreparedness. Feelings of guilt were the most common emotion preventing disclosure. Caregivers described feelings of guilt at having infected their children with HIV. The in-depth interviews results concurred:

“*Because I feel ashamed when I look at her always neh*. *And I was having that thought how am I going to tell her*” (*caregiver of participant 006*, *Interview*)

Anxiety was only overtly observed in one caregiver who appeared nervous about the child learning of her (the caregiver’s) status.

Thirty-three percent of caregivers felt the need to be emotionally prepared before disclosing to their children. This ranged from one caregiver not feeling “ready” to broach disclosure to another who was still struggling with her own HIV diagnosis. When presented with the process of disclosure during the pre-disclosure (screening) visit, these caregivers showed avoidance.

Two other caregivers had only discovered their child’s HIV status at a later age. The children’s medical records indicate perinatal exposure to HIV. These caregivers appeared to be in denial that their children were living with HIV. They were not emotionally prepared to address disclosure with their children and later discontinued the study.

### 2. Reasons why caregivers chose to disclose through the intervention

During the in-depth interviews, the caregivers were asked what prompted them to participate in the Disclosure study. Two sub-themes were evident when analysing their answers:

2.1 To explain why the child was taking medication
2.2 The need for assistance in disclosing and normalising the situation

#### 2.1 Explanations for why the child was taking medication

All participants in the disclosure study were on ARV treatment. Out of the 15 caregivers interviewed, nine (60%) expressed that their children were asking why they were taking medication.

“*The reason I brought him to disclosure is that maybe he will get information about why he has to drink the pills and further tell him his HIV status*” (*caregiver of participant 005*, *interview*)

The lack of disclosure had led to caregivers misleading their children about the necessity for the medication. Caregivers told their children that they were taking medication for ailments such as Asthma, Bronchopneumonia, TB, Bronchitis, and Tonsillitis, and for the flu. Additional reasons included that they were health supplements and vitamins which would keep them from falling down often, getting injured, and to help them grow bigger and/or stronger. The caregivers also commented that, at times, their children would not adhere to the medicine regime and “lie” about having taken their medication

“*she would give me stories/excuses when she was supposed to take her medication*” (*caregiver of participant 018*, *interview*).

#### 2.2 The need for assistance in disclosing and normalising the situation

Seven of the 15 caregivers interviewed (47%) expressed a need for assistance from more knowledgeable sources when disclosing to their children. In particular, the healthcare providers would be trusted to provide the necessary objectivity, support and information, and have the ability to normalise the situation.

“*I also needed some external help*, *if I can say so*, *to make him feel there is nothing wrong with whatever is happening or whatever is going on … I needed someone to assure him*, *besides me*, *to assure him that all’s good and that he’s good*.” (*caregiver of participant 001*, *interview*)

By normalising the situation, the healthcare providers were able to put into words the fears and apprehensions the caregivers were experiencing. Through their understanding, support and ability to educate the caregivers, the caregivers were able to model their own interactions through their observations of those of the healthcare providers. This improved their ability to address disclosure with their children. Furthermore, the acceptance of status by both the healthcare providers and caregivers, the acknowledgement of the caregiver’s status, as well as the knowledge that being HIV positive is not a death sentence or something to be ashamed about, assisted the child in accepting their status.

### 3. The caregivers’ response to the disclosure program

Throughout the study, the healthcare providers recorded their observations of how the caregivers responded to the various stages of the disclosure program on the DCFs

#### 3.1 Caregivers’ response pre-disclosure

The response was perceived by healthcare providers to be both negative and positive with six caregivers (20%) reported to be anxious, and five (17%) being hesitant. On the other hand, five (17%) expressed their happiness and appreciation to be part of the process.

#### 3.2 Caregivers’ response during full disclosure

Negative responses were more evident than positive ones with the most common being anxiety or unease (20%).

“*Granny was a bit anxious of her grandson’s reaction and was also assured*” *(caregiver of participant 003*, *Week 48 Visit*)

One caregiver’s anxiety appeared to be counter-productive and had a negative impact during the session. Instead of being calming and encouraging, the caregiver’s own anxiety resulted in attention being deflected from the participant to that of the caregiver at a crucial stage of disclosure.

“*Mom was in the session very anxious and at the same time trying to comfort her but a bit destructive” (caregiver of participant 015*, *Week 48 Visit*)

However, some caregivers responded positively by encouraging and reassuring the child.

“*The mom really helped by encouraging her to talk” (caregiver of participant 011*, *Week 48 Visit*)

#### 3.3 Caregivers’ response post-disclosure

At their post-disclosure visits, the caregivers had more positive (23%) than negative responses (7%), including satisfaction with their child’s progress.

“*Mom very happy with her school progress and even at home she’s coping very well” (participant 006*, *Week 72 Visit*)

Others reported relief that their children knew their status. One felt that the disclosure study was so helpful that she even decided to disclose to her other child. Other observations made included improved dyad relationships, understanding the importance of being responsible for the administration of medication and good nutrition. However, two caregivers (7%) appeared to respond negatively. These caregivers were observed as being emotionally distant. However, this was not explored further by the healthcare providers as the focus of the session was on the child participant.

“*No conversation after full disclosure as parents are not staying with the child…No one took the time to sit with her and talk*, *especially the mom (participant 026*, *Week 72 Visit*)

## Discussion

This study describes caregivers’ emotional response to a disclosure process, which assisted them in disclosing their children’s HIV+ status to them. Caregivers indicated their reluctance to disclose to their children. However, they were motivated to disclose during this study as medication adherence was becoming problematic and they felt more comfortable with a healthcare provider present to assist with disclosure and normalise the situation. Initially caregivers presented as being both anxious and grateful for the study. As the study progressed to full disclosure, many caregivers reacted negatively, with heightened anxiety. Following full disclosure, feelings of relief and satisfaction with their child’s progress was noted. This study also empowered one caregiver, post-disclosure, to independently disclose to her other child.

The caregiver’s attitudes towards HIV and HIV disclosure is shaped by their personal experiences within the environment and the attitudes of those around them, as surmised by social learning theory [19]. Our results illustrate the complexity of emotions experienced by the caregivers’ that perpetuated their reluctance to disclose to their children. This study allowed disclosure to occur in a contained environment under the nurturing and non-judgemental guidance of the healthcare provider. Healthcare providers modelled positive behaviours for both caregiver and child during disclosure, and guided the caregiver on how to disclose. This made the process less stigmatising and open, and provided a safe space, which fostered trust. The study reinforced perceptions that HIV should not be stigmatised and allowed caregivers space to gain confidence in their ability to manage disclosing to their children, as well as in talking about their own status.

The healthcare providers’ ability to model healthy attitudes towards HIV and reduce negative emotions around disclosure, taught caregivers how to model the same behaviour for their children. This was especially evident in reflections from the caregivers where they dealt with their own HIV status during the disclosure study. By applying social learning theory, addressing the negative attitudes and stigmatization held within the wider community the study could shift the caregiver’s experience of their own disclosure, reduce their anxiety and shame, and thus encourage more positive responses towards HIV self-disclosure [12, 35, 37]. Through this, children may learn not to fear the disease, and have less adverse responses following disclosure.

Initially, many caregivers responded to the intervention with anxiety. This is consistent with other studies [10, 20, 25, 35, 36, 38–40] and indicates a need to address the stigma still highly present in our communities that contributes to secrecy around HIV. The fear of stigma from others also leads to self-stigmatisation, making it difficult for caregivers to accept their own diagnosis, and in turn projecting that unacceptance and stigma onto their child [13, 25, 35]. This emphasises the need to equip healthcare providers to prepare and support caregivers throughout the disclosure process [20, 21, 41].

Early research reflected the fear of dying, and the portrayal of HIV-related deaths in the media as a barrier to disclosure [38, 39]. However, medical advancements, wider accessibility to ART, as well as the continued support our caregivers received from the PHRU Wellness Clinic, may explain why this was not reported in our study. Importantly, the two caregivers who only discovered their child’s status when the child was admitted to hospital for illness discontinued the study. This emphasises the need to ensure that caregivers are properly prepared, feel supported and have accepted their own diagnosis and that of their child before disclosure. It also stresses a need for community education to reduce the spread of misinformation and stigmatisation [19, 37, 42].

The child’s non-adherence to treatment was the most prominent motivation for caregivers to disclose in this study. Prior to disclosure, caregivers would mislead their children about the need for medication and deflect diagnosis to maintain adherence. However, caregivers noted that these strategies were losing effect and realised the need to disclose. There was also a tendency for some of the child participants in the study to “lie” about having taken their medication. Despite honesty being a desired and expected value in society, “white lies” and the “misleading” of other people is common [43, 44]. A “lie” serves the purpose of managing interpersonal relationships for personal gain or to avoid negative consequences [45]. Social learning theory postulates that observation, modelling and imitation are the cornerstones of social behaviour [19, 43, 44]. Therefore, it is not surprising that the caregivers’ resorted to misleading their children in their need for medication as they were attempting to ensure the child’s health. On the other hand, the children who chose to “lie” about taking their medication were attempting to avoid the negative consequences of their behaviour.

Social learning theory [19] suggests that a caregiver’s ability to talk openly and honestly about a diagnosis of HIV could have a positive effect on adherence in children as they will model more positive behavior. This then provides a powerful argument for the importance of shifting perceptions of shame and guilt around being diagnosed with HIV in caregivers. By empowering them, thus empowering their children living with HIV and improving the management of their illness.

This study showed that following full-disclosure and the improvement in “honesty” in the child-caregiver dyad, improved adherence was noted in several cases. This, with subsequent improvement in the child’s wellbeing, is in keeping with previous findings [37, 45–48]. However, other studies described both caregivers and children as expressing feelings of sadness following disclosure as they tended to perceive the child as being unable to be loved, eventually have sexual partners and/or having children uninfected with HIV of their own [22, 28].

The need to normalise the child’s HIV status was another prominent motivating factor for disclosure. This concurs with studies that reported that despite the fear caregivers have around disclosing to their children, they acknowledged that their children deserved the right to know their status [46, 48]. However, some studies reported that caregivers would prefer the children to find out their status on their own or at a much later age when the risk of them accidently disclosing their status was minimal [35]. Caregivers in this study stated that they required the assistance of the more knowledgeable healthcare providers in disclosing and normalising HIV to their children. Similarly, studies have found that most caregivers either preferred healthcare providers to disclose to their children, or required support from knowledgeable healthcare providers during disclosure [4, 46, 45]. However, other studies have reported that healthcare providers can also be viewed as placing pressure on caregivers to disclose [20, 49]. This emphasises the significant role healthcare providers play in the disclosure process.

Given the barriers experienced by the caregivers prior to disclosure, an initial negative response early in the disclosure process is not surprising. However, this sample of caregivers had an almost equal number of negative and positive responses towards disclosure initially. Post-disclosure found caregivers responding mostly positively with satisfaction and relief to the process. A sense of improved confidence, HIV knowledge and caregiver-child relationships were observed. Healthcare providers played a critical role in supporting the caregiver during disclosure, alleviating their anxieties and building their confidence. Furthermore, improved communication also assisted caregivers in becoming more comfortable talking about their own HIV status. Studies have reported that caregivers felt that a disclosure program that assisted them in developing more self-efficacy, an adequate knowledge base and provided emotional support, equipped them to better answer questions from their children and to handle the disclosure process [37, 42, 50–52]. Research has nevertheless identified some negative consequences of disclosure [26]. It was noted that some caregivers experienced increased stress when their children reacted negatively to disclosure [22]. This study, however, does not mention the timeline for when the results were obtained. Therefore, it is possible that these feelings of stress would have dissipated as time went on.

Limitations in this study included: 1) the positive responses may reflect a sampling bias, as these caregivers were willing and open to participate in both the main study and the sub-study. This could indicate a comfort and familiarity with the HIV clinic staff as most had been attending the clinic prior to the study. 2) Caregivers may have also provided socially desirable answers during the in-depth interviews due to familiarity with the interviewers from interactions during regular clinic visits. 3) A wide range of emotional responses were also not captured in the DCFs, a limitation when using observational reports. However, the in-depth interviews that followed the disclosure program, did allow the researchers to gain further insight into some of the DCF observations. 4) Caregivers who were observed as being distant following full-disclosure were not part of the in-depth interviews and their perception of the study may have been valuable. 5) This study has a predominant female perspective as most of the experiences and views were of female caregivers elicited through interviews with female researchers, and the DCF’s were completed and analysed by a female team. However, in many interventions mothers are often the caregivers accompanying the child to the HIV interventions. Regardless of these limitations, this study has added to the very limited literature regarding caregiver’s reactions during disclosure, especially in South Africa where the largest population of people living with HIV reside.

In keeping with social learning theory [19], caregivers own experience around HIV and disclosure is a process that parallels that of the child’s disclosure. Future studies should examine the impact of the caregiver’s own disclosure experience on their attitude towards their child’s disclosure. They should also explore at what age children start asking about taking medication, as this could be an indicator for when partial and full disclosure should be initiated. Understanding the reservations and motivations caregivers have towards disclosure can make an impact in shaping future HIV disclosure interventions. Implementing the 6 session disclosure program over a shorter time frame in clinics is not only possible, but also sustainable. With training in place, the sessions can be conducted by nursing staff, HIV counsellors or social workers. The sessions themselves are time consuming and require frequent visits by the caregiver and child, which may lower attendance to all sessions. However, the flexible nature of the program does allow clinic to accommodate both the caregivers’ availability and the children’s schooling schedules.

Many disclosure studies have focused on the children and not their caregivers. This study suggests the need to design interventions with support structures, in the form of healthcare providers, in place that assist the caregiver throughout the disclosure process; from the initial pre-disclosure session, during and following full disclosure to the child. Moreover, positive behaviours and attitudes modelled by healthcare providers play a significant role in assuring the caregiver during disclosure, and can start alleviating the effects of stigma from the community. This study has shown that a structured disclosure program with trained healthcare providers and supported caregivers is not only possible but is also a necessity in empowering children living with HIV. Therefore, the need for caregivers to be educated on the benefits of disclosure and risks of non-disclosure, as well being empowered to be open about their own diagnosis was highlighted.

## Supporting information

S1 File (DOCX)
