# Supplementary material for: Experiences of South African caregivers disclosing to their children living with HIV: Qualitative investigations
Source: PLoS One. 2022 Nov 29;17(11):e0277202. doi: 10.1371/journal.pone.0277202 (PMC9707749; doi:10.1371/journal.pone.0277202)
Supplement: S1 File — (DOCX) [file pone.0277202.s001.docx]

**Supplementary Table 1: Caregiver – Child Participant Relationship**

| **Participant** | **Relationship to participant** | **Education Level** | **Employment Status** | **HIV Status** | **Caregiver Receiving ARTs** | **Caregiver Interviewed** | **Child Age** | **Child Gender** | **Time of Full Disclosure** |
| --- | --- | --- | --- | --- | --- | --- | --- | --- | --- |
| 001 | Father | NQF-4 | Employed | Negative | N/A | Yes | 8 | Male | Week 72 |
| 002 | Mother | Grade 11 | Employed | Positive | Yes | Yes | 10 | Female | Week 72 |
| 003 | Grandmother | Grade 5 | Employed | Positive | Yes | No | 11 | Male | Week 48 |
| 004 | Mother | Grade 9 | Unemployed | Positive | Yes | Yes | 10 | Female | Week 48 |
| 005 | Mother | Grade 9 | Unemployed | Positive | No | Yes | 8 | Male | No |
| 006 | Mother | Grade 11 | Unemployed | Positive | Yes | Yes | 11 | Female | Week 48 |
| 007 | Mother | Grade 12 | Employed | Positive | Yes | No | 10 | Male | Week 36 |
| 008 | Mother | Grade 11 | Employed | Positive | Yes | No | 10 | Female | Week 48 |
| 009 | Aunt | Grade 11 | Unemployed | Positive | Yes | No | 9 | Female | Week 12 |
| 010 | Mother | Grade 11 | Unemployed | Positive | Yes | No | 10 | Female | Week 48 |
| 011 | Mother | Grade 11 | Employed | Positive | Yes | No | 11 | Female | Week 48 |
| 012 | Mother | Grade 12 | Unemployed | Positive | Yes | No | 8 | Female | Week 72 |
| 013 | Mother | NQF-4 | Unemployed | Positive | Yes | No | 11 | Female | Week 48 |
| 014 | Grandmother | Grade 11 | Unemployed | Positive | Yes | No | 9 | Female | Week 72 |
| 015 | Mother | Grade 10 | Employed | Positive | Yes | No | 11 | Female | Week 48 |
| 016 | Mother | Auxiliary Nursing | Unemployed | Positive | Yes | Yes | 9 | Female | No |
| 017 | Mother | Grade 12 | Unemployed | Positive | Yes | Yes | 12 | Female | Week 36 |
| 018 | Mother | Grade 8 | Unemployed | Positive | Yes | Yes | 10 | Female | Week 72 |
| 019 | Mother | Grade 10 | Unemployed | Positive | Yes | Yes | 11 | Male | Week 48 |
| 020 | Mother | Grade 12 | Unemployed | Positive | Yes | No | 12 | Male | Week 48 |
| 022 | Father | Grade 12 | Unemployed | Negative | N/A | No | 11 | Male | No |
| 023 | Mother | Grade 11 | Unemployed | Positive | Yes | Yes | 11 | Male | Week 48 |
| 024 | Mother | Grade 12 | Unemployed | Positive | Yes | No | 9 | Male | Week 72 |
| 026 | Father | Unknown | Employed | Positive | Yes | No | 11 | Female | Week 48 |
| 027 | Mother | Grade 12 | Unemployed | Positive | Yes | No | 10 | Female | Week 48 |
| 028 | Mother | Grade 12 | Employed | Positive | Yes | Yes | 11 | Male | Week 48 |
| 029 | Mother | Grade 9 | Unemployed | Positive | Yes | Yes | 11 | Female | Week 48 |
| 030 | Mother | Grade 12 | Unemployed | Positive | Yes | Yes | 12 | Male | Week 72 |
| 031 | Mother | Grade 11 | Unemployed | Positive | Yes | Yes | 10 | Male | Week 48 |
| 032 | Mother | Grade 11 | Employed | Positive | Yes | Yes | 11 | Female | Week 48 |

**Supplementary Table 2: Barriers to Disclosure taken from the Disclosure Counselling Forms**

| **Sub-Theme** | **Code** | **Examples from data** |
| --- | --- | --- |
| 1.1 Caregiver practically ill-equipped | a. Lack of Confidence in HIV Knowledge | “father said he was not sure what to say to the child; not sure what information to give” (001, Screening Visit)  "She didn't know what to say to the child and scared that she won't be able to answer the questions" (013, Screening Visit) |
|  | b. Child not old enough for disclosure | “The mom felt she was too young, her daughter” (002, Screening Visit) |
| 1.2. Fear | Stigma | “There are family dynamics that she still fears about her HIV status. It looks like she has never really come to terms with her HIV status (004, Week 24 Visit)  “mom talked a lot about stigma and the fact that she’s never disclosed to anyone except her partner who rejected her afterwards” (015, Screening Visit)  “Her child can’t keep a secret, so she is afraid that she will tell everybody” (028, Screening Visit) |
|  | Fear in itself | “Mom feels the child will run way” (004, Week 12 Visit)  “Caregiver expressed her fears and asked a lot of questions” (007, Screening Visit) |
|  | Rejection | “mom f;l0=[-peared rejection” (003, Screening Visit) |
| 1.3 Caregiver Lack of Emotional readiness | Not emotionally ready | “Mom has never been supported about her own status so as a result she’s not really comfortable talking about HIV” (027, Screening Visit) |
|  | Guilt | “mom didn’t want to burden her son with her guilt and weight of being HIV+” (007, Week 12 Visit)  “mom is burdened with the guilt that she infected the child” (015, Screening Visit)  “she felt guilt, of all her children, why him” (031, Screening Visit) |
|  | Avoidance | “The dad seemed not to be free talking about HIV” (026, Screening Visit)  “Mom has never been supported about her own status so as a result she’s not really comfortable talking about HIV” (027, Screening Visit) |
|  | Anxiety | “mom seemed nervous about the child knowing her status” (004, Week 12 Visit) |
|  | Only found out Recently | "The mom found out last year when the child was hospitalised about her status. The child since started ARVs and she's curious about why she is taking meds (016, Screening Visit)  "The dad just found out last year November about his son's status, as he was admitted in hospital. The child started ARV treatment" (022, Screening Visit) |

**Supplementary Table 3: Caregiver’s observed reaction to the Disclosure Process taken from the Disclosure Counselling Forms**

| **Subtheme** | **Code** | **Examples from Data** |
| --- | --- | --- |
| 3.1 Pre- Disclosure | **Negative Responses**  a. Anxiety | “Father was told not to panic, and that he will be supported by the staff” (001, Week 24 Visit)  “Mom seemed nervous about the child knowing her status…Mom feels that the child will run away” (004, Week 12 Visit) |
|  | b. Hesitancy/ Avoidance | “The father said he is not ready to let the child know about HIV because he is not sure the child will comprehend the information” (001, Week 24 Visit)  “Mom has been avoiding any subject that related to HIV (002, Week 48 Visit) |
|  | **Positive Responses**  a. Happy | “mom says she’s seen a big improvement in her child” (017, Week 12 Visit)  “Mom is happy with progress” (005, Week 12 Visit) |
|  | b. Satisfaction with program | “Father is happy about the process and promised to support the child” (001, Screening Visit) |
|  | c. Relief | “Mom relieved about the process” (014, Screening Visit) |
| 3.2. Full Disclosure | **Negative Response**   1. Anxiety/ Uneasy | “Granny was a bit anxious of her grandson’s reaction and was also assured” (003, Week 48 Visit)  “mom was the one feeling uneasy specially after disclosing, she kept on doing disturbing mannerisms” (010, Week 48 Visit) |
|  | b. Counter-productive assurance | “Mom was in the session very anxious and at the same time trying to comfort her but a bit destructive” (015, Week 48 Visit) |
|  | **Positive Response**   1. Encouragement/Reassurance | “The mom really helped by encouraging her to talk” (011, Week 48 Visit)  “Tried to reassure him” (028, Week 48 Visit) |
| 3.3. Post -Disclosure | **Negative Response**  a. Emotionally distant | “Father seemed distant” (013, Week 72 Visit)  “No conversation after full disclosure as parents are not staying with the child…No one took the time to sit with her and talk, especially the mom (026, Week 72 Visit) |
|  | **Positive**   1. Satisfied/ Happy | “Mom very happy with her school progress and even at home she’s coping very well” (006, Week 72 Visit) |
|  | b. Prompted further disclosure | “disclosure study helped her that she even decided to disclose to her older daughter cos (sic) she is HIV+ve too” (008, Week 72 Visit) |
|  | c. Relief | “mom was very anxious but she seemed relieved about her daughter after disclosure” (029, Week 48 Visit) |
